# Case Report: COVID-19 Infection and Cervical Artery Dissection

**DOI:** 10.4269/ajtmh.21-0999

**Published:** 2022-01-24

**Authors:** Kaylynn Purdy, Rebecca Long, Glen Jickling

**Affiliations:** Department of Medicine, Division of Neurology, University of Alberta, Edmonton, Canada

## Abstract

A 45-year-old woman presented 3 days after symptom resolution from a COVID-19 infection with a left vertebral artery dissection with no known preceding trauma or underlying disposition. She subsequently suffered a left lateral medullary stroke 15 hours after her initial presentation. Cervical artery dissections (CeAD) can occur in the absence of trauma, and in some cases, infection may be a contributing factor. COVID-19 infection can cause an endotheliopathy and inflammatory response, which may contribute to intimal vessel disruption. Whether COVID-19 infection can contribute to CeAD and subsequent stroke is discussed, along with other considerations regarding the pathogenesis of CeAD.

## INTRODUCTION

In March 2020, the WHO declared COVID-19, a disease caused by the SARS-CoV-2 virus, a pandemic. By May 19, 2020, there were just under 5 million cases described, and more than 300,000 deaths worldwide.^1^ Although the disease causes primarily respiratory pathologies, there are increasing accounts of neurologic sequelae.^1^ Here, we discuss a case of a previous healthy individual who presented with a cervical artery dissection (CeAD) in the context of a recent COVID-19 infection.

## CASE REPORT

**Figure 1.**
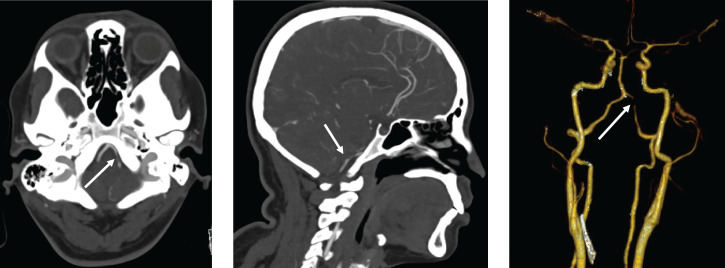
Computed tomography angiogram showing narrowing and irregularity of the left V4 segments of the vertebral arteries (white arrows). This figure appears in color at www.ajtmh.org.

**Figure 2.**
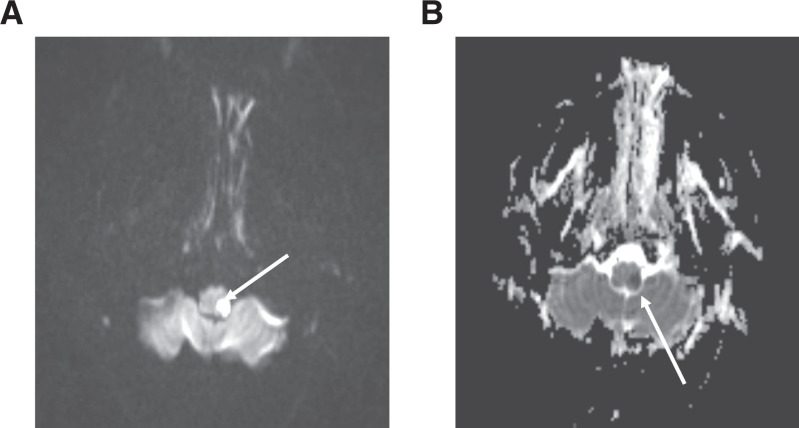
Brain magnetic resonance imaging scan showing infarct in the left lateral medulla with hyperintensity on Diffusion-weighted imaging (**A**) and hypodensity on apparent diffusion coefficient (**B**) (white arrow).

A 45-year-old female of South Asian ethnicity with history of migraines contracted COVID-19 from a workplace exposure. She did not have history of hypertension, diabetes, or cigarette smoking. She had mild symptoms of COVID-19 with cough and fever not requiring hospitalization or treatment. Her symptoms resolved completely within 10 days. However, 3 days after symptom resolution she developed a sudden-onset occipital headache, along with transient left facial paresthesia and mild left-sided weakness. A brain computed tomography (CT) was performed that was unremarkable; however, her CT angiogram (CTA) revealed a left V4 vertebral artery dissection (Figure 1). She was loaded with 325 mg of acetylsalicylic acid. Fifteen hours later, she had a sudden decline in neurologic function with severe vertigo, nausea, and vomiting. She was found to have hypophonic and hoarse speech, deconjugate gaze with direction changing nystagmus worse on right gaze, mild left facial weakness, left arm and leg weakness Medical Research Council (MRC) grade 4/5, left arm and leg ataxia, and mildly decreased pin-prick sensation on her left face and right arm and leg. Her NIH Stroke Scale score was 8. During her admission, the patient had a peak leukocyte count of 17.2 × 10^9^ cells/L, with an associated increase in neutrophils (15.2 × 10^6^ cells/L). Repeat CTA showed a persistent narrowed left vertebral artery, and no infarct was evident on brain CT. A brain magnetic resonance imaging scan revealed restricted diffusion in the left lateral medulla consistent with acute infarction (Figure 2). The patient’s syndrome was consistent with an acute left lateral medullary syndrome, also known as Wallenberg syndrome or posterior cerebellar artery syndrome, with likely involvement of the pyramidal decussations accounting for the ipsilateral weakness.

The patient was outside the window for thrombolysis and no large vessel occlusion was present for endovascular therapy. Anticoagulation with heparin was considered. However, because there was no clear thrombus and the dissection was intracranial, anticoagulation was generally contraindicated because of the increased risk of bleeding, including subarachnoid hemorrhage with intracranial dissections.^2^ It was decided that acetylsalicylic acid 81 mg daily was the safest option, and it was continued for secondary stroke prevention. She gradually improved in hospital but continued to have persisting difficulty with dysphagia, diplopia, and left-sided weakness, requiring several months of rehabilitation. At an 11-month follow-up, she had near complete recovery from her deficits with only minor gait instability remaining.

## DISCUSSION

Dissection occurs when blood tracks between layers of the vessel wall, with hematoma separating the inner tunica intima layer from the middle tunica media or outer tunica adventitia.^3^ Stroke occurs from CeAD either from thromboembolism from vessel wall irregularities or hypoperfusion due to vessel stenosis.^3^ The most common symptom of CeAD is headache, which often is frontal or periorbital in carotid dissection and occipital in vertebral dissection.^3^ Neck pain and Horner syndrome are also common features.

Trauma is a potential cause of CeAD, with minor trauma history elicited in up to 40% of cases of cervical dissections.^4^ However, a majority of minor trauma that occurs over the course of a person’s lifetime is not associated with CeAD; thus, additional factors may be required for dissection to occur.^4^ Connective tissue disorders do increase the risk for dissection; these disorders include Ehlers-Danlos syndrome, Marfan syndrome, and fibromuscular dysplasia.^3^ In the case discussed here, the patient had only a mild cough with no known trauma, and no signs of connective tissue disease. Currently there are two other cases reported in the literature of CeAD occurring in the setting of COVID-19 infection: one of bilateral CeAD in an otherwise healthy 39-year-old woman, and an additional case report of a previously healthy 38-year-old woman with left common carotid dissection.^5^^,^^6^ The temporal association of our patient’s COVID-19 illness, and that of the two other cases reported, raises the question as to whether aspects of infection and inflammation may have contributed to spontaneous dissection.

Infection may increase susceptibility to dissection.^7^ The associated inflammatory process may promote vessel injury and raise the risk of dissection.^7^^,^^8^ Infection alone in the absence of mechanical factors (e.g., cough, sneezing, vomiting) has been associated with CeAD.^9^ Patients with nontraumatic (spontaneous) CeAD often have elevated leukocyte counts compared with traumatic dissections, as seen in the patient presented in this case study.^10^ CeAD also peaks seasonally during the autumnal months, which further supports an infectious association.^9^ Even in the absence of a known infection, an inflammatory process causing a transient arteriopathy may explain aspects of CeAD, the low recurrence rate, and prevalence of simultaneous dissections in separate vessels.^9^

COVID-19 is a systemic infection with a broad range of clinical manifestations, from fever and cough to acute respiratory distress and multiorgan failure.^11^ Several potential mechanisms exist by which COVID-19 infection may enhance susceptibility to CeAD.

In a dissection, the intima, which comprises endothelium and connective tissue including fibrillin-1, collagen, and elastic fibers, is disrupted. Each of these components may be affected by COVID-19 infection, potentially increasing the risk of dissection. Endothelial cells can be affected by SARS-CoV-2 virus either directly through the angiotensin converting enzyme-2 receptor or indirectly through inflammatory activation. This can produce an endotheliopathy that may contribute to intimal disruption and dissection.^12^

COVID-19 may also disrupt connective tissue in the intimal layer. Fibrillin-1 has important roles not only in maintaining connective tissue integrity but also in regulation of growth factors. *FBN1* is the gene mutated in Marfan syndrome, leading to dysfunctional fibrillin-1. Whether COVID-19 alters fibrillin-1 remains unknown. A function of fibrillin-1 is to bind and sequester the cytokine transforming growth factor-beta (TGF-β). COVID-19 does increase TGF-β, which may alter the dynamics of TGF-β–fibrillin-1 binding, promoting connective tissue disruption.^13^^,^^14^ Loeys-Dietz syndrome highlights the importance of TGF-β, where a TGF-βR2 mutation results in a Marfanoid-type syndrome, including increased risk of arterial dissection.^15^

Collagen and elastic fibers may also be affected by COVID-19. Disruption of collagen can weaken a blood vessel and predispose to dissection. In Ehlers-Danlos syndrome, mutations in collagen such as COL3A1 increase the risk of dissection.^16^ In COVID-19, inflammation may lead to the generation of metalloproteinases or other collagenases released by activated immune cells, leading to transient disruption of collagen. Likewise, elastic fibers may also be degraded by elastases and other proteases released during inflammation induced by COVID-19.

## CONCLUSION

We report a patient with vertebral artery dissection causing lateral medullary infarction in the setting of a recent COVID-19 infection. The role of COVID-19 in CeAD remains unclear. Although further study is needed, COVID-19 may increase susceptibility to CeAD through several potential mechanisms, including cough, endotheliopathy, and inflammatory disruption of connective tissue in the intimal layer. Patients with a COVID-19 infection should be carefully monitored for dissection and endothelial disease, and with the appropriate clinical signs, including new headache, neck pain, and neurological deficits, suspicion for a possible CeAD is warranted.
